# Psychosocial profiles influencing healthy dietary behaviors among adolescents in Shandong Province, China: a cross-sectional study

**DOI:** 10.3389/fnut.2024.1418950

**Published:** 2024-09-19

**Authors:** Ya Shi, Lin Fu, Shengping Li, Ke Jiang, Zumin Shi, Manoj Sharma, Yong Zhao

**Affiliations:** ^1^School of Public Health, Chongqing Medical University, Chongqing, China; ^2^Research Center for Medicine and Social Development, Chongqing Medical University, Chongqing, China; ^3^Research Center for Public Health Security, Chongqing Medical University, Chongqing, China; ^4^Nutrition Innovation Platform-Sichuan and Chongqing, School of Public Health, Chongqing Medical University, Chongqing, China; ^5^Chongqing Health Center for Women and Children, Women and Children’s Hospital of Chongqing Medical University, Chongqing, China; ^6^Human Nutrition Department, College of Health Sciences, QU Health, Qatar University, Doha, Qatar; ^7^Department of Social and Behavioral Health, School of Public Health, University of Nevada, Las Vegas, NV, United States; ^8^Department of Internal Medicine, Kirk Kerkorian School of Medicine, University of Nevada, Las Vegas, NV, United States; ^9^Chongqing Key Laboratory of Child Nutrition and Health, Children’s Hospital of Chongqing Medical University, Chongqing, China

**Keywords:** psychosocial profiles, dietary behavior, adolescent, Shandong, cross-sectional study

## Abstract

**Objectives:**

We aimed to assess the influence of psychosocial profiles on dietary behaviors among school-aged adolescents in China.

**Methods:**

A cross-sectional study was conducted involving 7,862 adolescents from 100 schools in Shandong, China. Psychosocial profiles and dietary behaviors were assessed using the Junior High School Students’ Psychosocial Profiles Questionnaire (JPPQ) and the Chinese Diet Quality Questionnaire (DQQ), respectively. Linear regression models were used to investigate the association between adolescents’ psychosocial profiles and dietary behaviors.

**Results:**

The mean age of the participants was 13.18 ± 1.15 years; 48.5% of them were boys. The majority of participants (97.90%) were Han Chinese, and approximately half of the participants (50.90%) resided in rural areas. After adjusting for sociodemographic characteristics and family computer and Internet ownership and usage, healthy dietary behavior was positively correlated with higher psychosocial profile scores (*p <* 0.05). The stratified analysis results revealed that the group with the highest psychosocial profile score was associated with an increased overall global dietary reference (GDR) score in “households without a family computer and Internet” ( β : 5.357, 95% Cl: 4.931–5.784, *p <* 0.05).

**Conclusion:**

Good psychosocial profiles exhibit a positive influence on healthy dietary behaviors. Therefore, policymakers should focus on Internet usage to maximize the positive effects on global youth health behaviors.

## Introduction

Adolescence is a critical transitional stage in life, during which individuals are particularly susceptible to psychological issues stemming from various factors, such as the school environment and social influences (1, 2). Emotional stressors such as academic pressure, exposure to bullying at school, and familial conflicts can contribute to the development of psychological disturbances in adolescents. Additionally, the complexities of interpersonal relationships during this period further exacerbate these problems (3). The widespread use of online media devices also increases the risk of sleep deprivation among adolescents, which can have detrimental effects on both their physical health and mental well-being (4). According to the World Health Organization, approximately one in seven individuals aged 10–19 years worldwide experiences mental disorders, accounting for 13% of the disease burden in this age group (5).

Psychosocial profiles of adolescents encompass a multifaceted construct that integrates societal and psychological elements. These profiles can be assessed through the adolescent’s self-reported internalizing indicators, such as self-perception, emotional self-awareness, and externalizing behaviors, including school bullying and aggressive tendencies. These indicators also reflect adolescents’ psychological status (6, 7). Research has shown that negative self-perceptions, lack of supportive peer relationships, and exposure to school bullying may contribute to the onset or exacerbation of depression (8–13). As adolescents progress through this stage of life, their physical and psychosocial development can significantly influence their food choices and eating behaviors (14, 15).

As adolescents seek independence and social connections, they may consume more calorie-dense foods, which can lead to obesity (16). Furthermore, social media exposure among children and adolescents promotes increased eating behaviors during viewing sessions, which also leads to obesity (17).

Consequently, these changes in psychological profiles and sedentary behaviors increase the risk of obesity among adolescents, which is becoming a significant public concern globally (18).

Given the abovementioned trend, the 2020–2025 Dietary Guidelines for Americans (DGA) emphasize the importance of establishing and maintaining healthy dietary behaviors early in life in order to reduce the risk of diet-related chronic diseases (19, 20). Evaluating dietary behaviors involves the use of dietary quality assessment tools to measure dietary quality scores, which help in determining adherence to a healthy dietary pattern (21, 22).

Healthy dietary patterns, generally characterized by the consumption of fruits, vegetables, fish, and whole grains, are widely recognized as essential components of a healthy diet. These patterns may reduce the incidence of mental illness and improve psychological well-being (23–26). Unfortunately, research by Chinese scholars indicates that the current eating habits of Chinese teenagers are suboptimal. A study involving 12,860 adolescents aged 11–18 years in Shanghai revealed that a significant portion of their diet consisted of high-sugar and high-calorie foods, with the prevalence of consuming vegetables and fruits less than once a day being 25.9 and 47.2%, respectively (27).

Additionally, a school-based investigation in Zhengzhou reported that up to 80.5% of vocational high school students consumed sugar-sweetened beverages at least once a week (28). There is a bidirectional relationship between psychological states and healthy dietary behaviors. Specifically, a positive psychological state often leads to better attention to dietary variety and balanced nutrition, thereby enhancing dietary quality. Similarly, a high-quality diet can help reduce psychological disorders. This reciprocal relationship underscores how improvements in one area can positively influence the other, creating a mutually reinforcing effect. Nutritional interventions may be enhanced by comprehending the psychological status of adult food consumers (29). As children grow older and face greater stress, the development of healthy eating behaviors becomes more constrained (30). These findings suggest that psychological status is intricately linked to healthy eating behaviors.

The majority of research on the correlation between healthy eating and psychosocial profiles has primarily concentrated on adults and the elderly, with limited investigation on younger adults. Furthermore, Rajaram et al. (31) and Parrott et al. (32) have highlighted the significant focus on memory cognition and memory disorders in various studies related to dietary patterns, with limited exploration of their connection to psychological status. Additionally, studies on the relationship between eating behaviors and different psychological status among adolescent populations are lacking.

Relevant reviews have emphasized the importance of addressing the long-term health outcomes of adolescents (33). With the rising prevalence of mental illness and psychosocial problems among secondary school students and the significant role of healthy dietary behaviors in maintaining well-being, it is imperative to investigate the impact of psychosocial profiles on the dietary behaviors of adolescents. To fully consider the psychosocial profiles of adolescents, we analyzed three main layers: self-perception, which is an internalization indicator, bullying perception, and peer relations, which serve as externalization indicators.

## Materials and methods

### Study design and sample

In this study, we utilized the data retrieved from the Population Health Data Archive (PHDA), which is a data-sharing platform. This cross-sectional study was conducted to investigate the impact of psychological status on health and health-related behaviors of junior school students in Shandong, China. The probability proportional to size (PPS) sampling method was adopted, and 100 schools from 10 administrative districts were randomly selected based on the specific geography, population, and socio-economic level of the province in the 2020 academic year. Schools with fewer than 100 students in each grade or fewer than 300 students in total were not included in this study. Information regarding socioeconomic status, social interaction, nutrition and diet, psychological status, mental health, school adaptation, quality of life, spare-time physical activity, risk behaviors, and physical fitness was obtained for this study.

**Figure 1:**
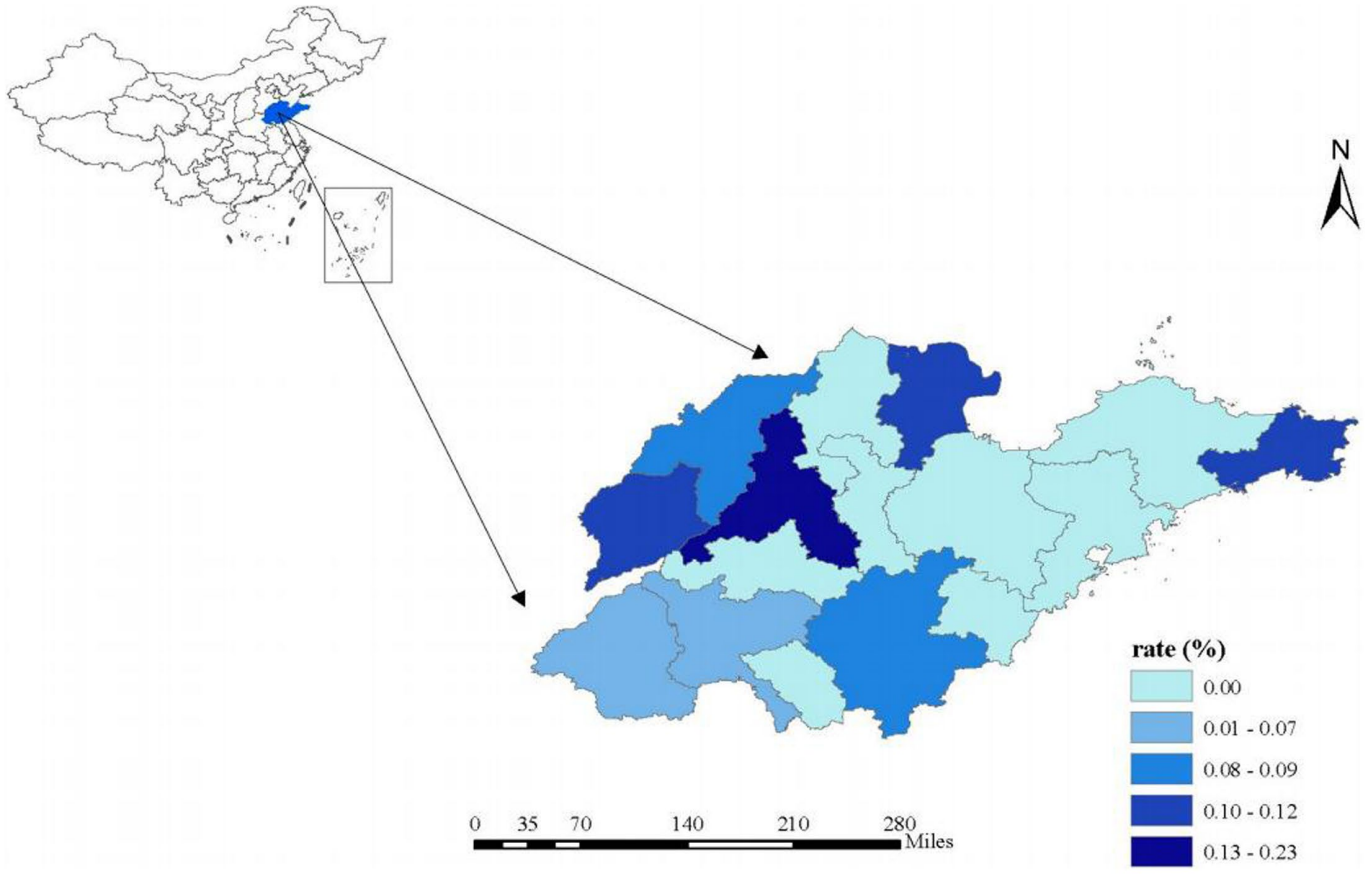
Distribution of survey respondents by city. The proportion of adolescents distributed in each city = the number of respondents in each city/the total number of respondents* 100%.

**Figure 2:**
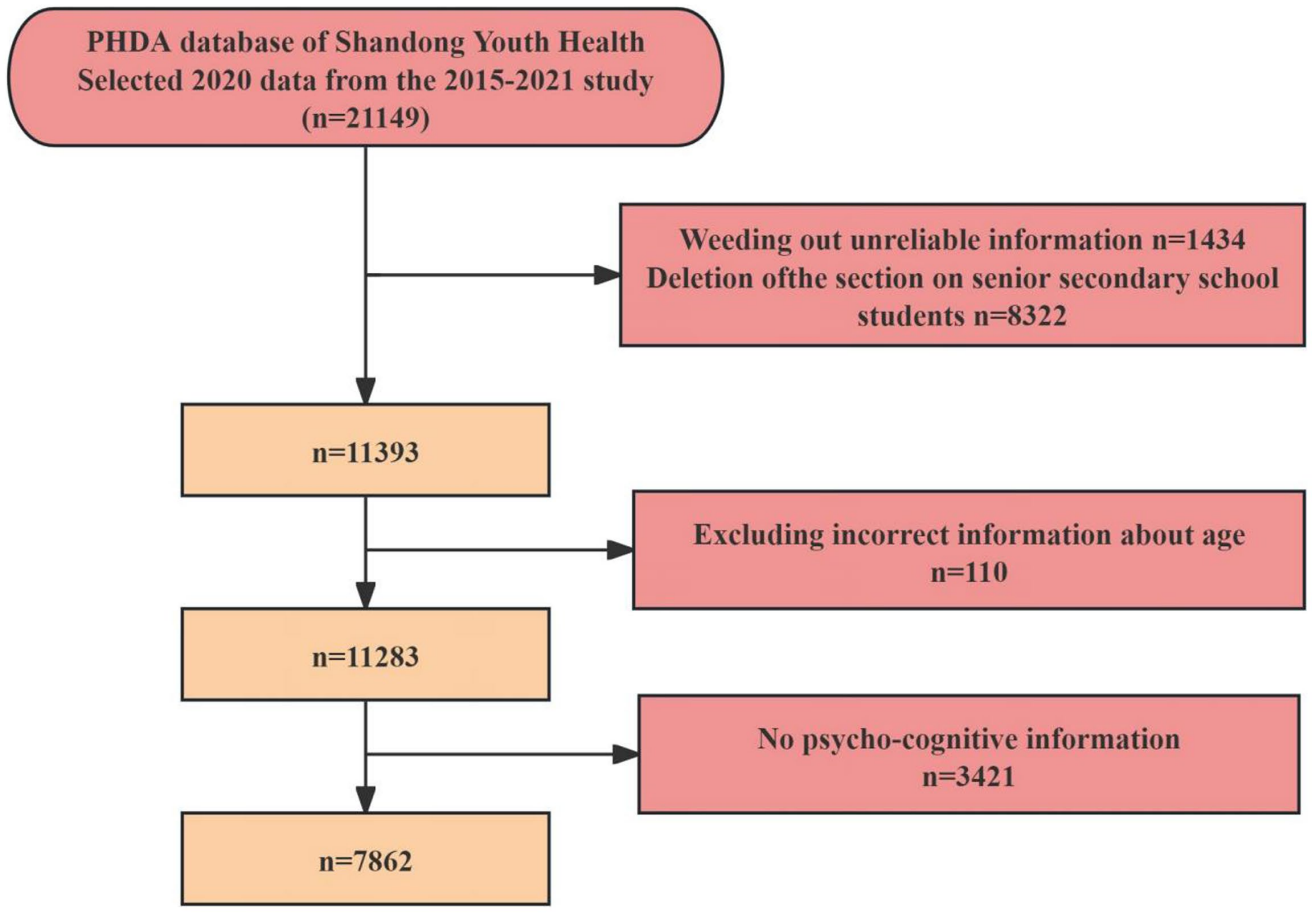
Flowchart of participant.

Further details of the PHDA design and sampling methods have been published in previous research (34, 35). The survey sample consisted of 11,393 junior adolescents from Shandong Province. After excluding unreasonable samples and those with missing information on crucial sociological characteristics or psychological status, a total of 7,862 participants were included in the data analysis. Our study encompasses cities from approximately half of the prefecture-level cities in Shandong, covering the three principal regions: Lunan, Luzhong, and Lubei. The sample distribution across these regions demonstrates variability, with each region contributing between 0.07 and 0.23% of the total sample. This distribution aligns with the observed trend of higher population density in the more developed areas and lower density in the less developed regions of Shandong. The sample was randomly selected from various cities across the Shandong Province, ensuring a comprehensive and representative view (Figures 1, 2).

### Outcome variables: junior high school students health and dietary patterns score

The 17-item questionnaire was developed based on the Global Dietary Reference (GDR) list. Participants’ dietary status was evaluated using the modified Chinese Diet Quality Questionnaire (DQQ), which is a 5-point scale (1–5) for rapid qualitative and quantitative analyses of participants’ diet quality (36). An additional inventory file shows this in more detail (Supplementary material S1).

The DQQ includes three dietary scores: the GDR healthy score, the GDR-limit score, and the overall GDR score. The GDR-healthy score reflects global recommendations for health-promoting foods in healthy diets. The GDR-limit score reflects global recommendations for limiting certain dietary components. A low overall GDR score, a low GDR-healthy score, and a high GDR-limit score indicate poor diet quality (34, 37). Frequencies of weekly consumption in the questionnaire were scored as follows: more than once a day (5), about once a day (4), about once every other day (3), one or two times a week (2), and never (1).

The total dietary pattern score was calculated based on the two components using the formula below: overall GDR score = GDR-Healthy score-GDR-Limit score. A high overall GDR score represents high quality. In this study, Cronbach’s *α* was 0.87, and the questionnaire demonstrated good construct validity (KMO 0.91, Bartlett’s test *p* < 0.001).

### Exposure variables: junior high school students’ psychosocial profiles

The Junior High School Students’ Psychosocial Profiles Questionnaire (JPPQ) has 17 items (Supplementary material S2). It has three main sections, such as self-perceptions, peer relationships, and perceptions of school bullying. The tool was developed based on the relevant psychological studies (35, 38). Using a 5-point scale, the JPPQ assessed participants’ psychological status, with acceptable internal consistency for self-perceptions, peer relationships, and school bullying perceptions (Cronbach’s *α* = 0.86) in this research. Each participant was asked to answer the self-perception questions such as “In general, I have a lot to be proud of,” with responses ranging from low to high across five levels, namely (1) never, (2) seldom, (3) sometimes, (4) usually, and (5) always. Additionally, they were asked to respond to the questions about peer relationships and bullying perceptions of the tool, which is a validated method for assessing psychosocial status. The students completed the questionnaire by responding to a series of inquiries. For example, “I enjoy studying with my friends,” with responses similarly divided into five levels, namely (1) completely inconsistent, (2) not quite consistent, (3) sometimes fit, (4) more in line with, and (5) very consistent. For negatively phrased items, the score assignments were reversed. For example, the item “I cannot talk with other people” had its scores reversed: (5) completely inconsistent, (4) not quite consistent, (3) sometimes fit, (2) more in line with, and (1) very consistent. Thus, the overall score of the psychosocial profiles was calculated by summing the scores of the self-perceptions, peer relationship perceptions, and campus bullying perceptions. A high overall score indicates a positive psychosocial status, reflecting a favorable trend in social skills and self-confidence.

### Covariates

Adolescents’ demographic information includes age, sex (boys/girls), residence, nationality (the Han nationality/minority), educational level of adolescents’ parents (primary school or below/secondary school/secondary vocational school, high school/college or higher), family wealth level (poor/middle/good), accommodation (no/yes), single son or daughter (no/yes), and residence (urban/rural). These variables, along with the family computer and network situation (none/ one of the above/both), were considered covariates in this study. The family computer and Internet situation was ascertained through self-reporting. Specifically, participants responded to the question, “Do you have a computer and internet access at home?” by selecting from the options of “none,” “one of the above,” and “both.” The classifications of these variables were predefined based on previous research (39, 40).

### Statistical analysis

All data were analyzed using SPSS (version 26.0, IBM Corporation, Armonk, NY, United States) and Stata software (version 18.0, Stata Corp., LLC). Categorical variables were described using frequencies and percentages, whereas the mean (standard deviation) was used to describe adolescents’ age and the different GDR scores (GDR-healthy, GDR-limit, and overall GDR). The scores were converted to percentages and then divided into quartiles to analyze the psychosocial profiles of adolescents. Categorical variables were compared using the chi-squared test, while continuous variables were analyzed using ANOVA to assess differences between groups. Linear regression analysis was employed to investigate the relationship between adolescents’ dietary patterns and their psychosocial profiles. In subgroup analyses, the multiplicative interaction between psychosocial profiles and covariates (sex, nationality, accommodation, residence, only child, family economic status, family computer and network situation, and parental education level) was examined by including the product of these variables in the regression model. All tests were two-sided, and a *p*-value of < 0.05 was considered statistically significant.

## Results

### Basic demographic characteristics

The sample characteristics are presented in Table 1. A total of 7,862 participants were included (48.50% were boys and 51.50% were girls). The majority of participants (97.90%) were Han Chinese, and approximately half of the participants (50.90%) lived in rural areas. The average age of participants was 13.18 ± 1.15 years. A small proportion of junior high school students (25.50%) were only children; similarly, only 29.40% of them chose to board at school from Monday through Thursday. Computer network penetration in the families of people in the high psychosocial profile subgroup was significantly higher than that in the low subgroup.

**Table 1:** Sample characteristics by quartile of adolescents’ JPPQ score: PHDA (*N* = 7,862).

Characteristics	Total		Adolescents’ JPPQ score^a^								*p*-value
			Quartile 1		Quartile 2		Quartile 3		Quartile 4		
Sample (*n*)	*N* = 7,862		*N* = 2,106		*N* = 1,897		*N* = 1,891		*N* = 1,968		
Sex (*n*, %)											0.206
Boys	3,810	(48.50)	1,048	(49.20)	889	(46.90)	896	(47.60)	977	(50.00)	
Girls	4,052	(51.50)	1,084	(50.80)	1,005	(53.10)	986	(52.40)	977	(50.00)	
Age, mean (SD)	13.18	(1.15)	13.36	(1.12)	13.19	(1.11)	13.09	(1.15)	13.05	(1.20)	<0.001^**^
Nationality (*n*, %)											0.479
Han	7,693	(97.90)	2,091	(98.10)	1,850	(97.70)	1,835	(97.50)	1,917	(98.10)	
Other	169	(2.10)	41	(1.90)	44	(2.30)	47	(2.50)	37	(1.90)	
Residence (*n*, %)											<0.001^**^
Urban	3,862	(49.10)	881	(41.30)	906	(47.80)	977	(51.90)	1,098	(56.20)	
Rural	4,000	(50.90)	1,251	(58.70)	988	(52.20)	905	(48.10)	856	(43.80)	
Only child (*n*, %)											<0.001^**^
Yes	2,002	(25.50)	481	(22.60)	411	(21.70)	503	(26.70)	607	(31.10)	
No	5,860	(74.50)	1,651	(77.40)	1,483	(78.30)	1,379	(73.30)	1,347	(68.90)	
Family wealth level (*n*, %)											0.03^*^
Poor	937	(11.90)	265	(12.40)	233	(12.30)	231	(12.30)	208	(10.60)	
Medium	6,325	(80.50)	1,674	(78.50)	1,536	(81.10)	1,516	(80.60)	1,599	(81.80)	
Good	600	(7.60)	193	(9.10)	125	(6.60)	135	(7.20)	147	(7.50)	
Father’s education level (*n*, %)											<0.001^**^
College or higher	1,381	(17.60)	246	(11.50)	268	(14.10)	403	(21.40)	464	(23.70)	
High school	2,484	(31.60)	546	(25.60)	627	(33.10)	622	(33.00)	689	(35.30)	
Junior high school	3,314	(42.20)	1,046	(49.10)	843	(44.50)	730	(38.80)	695	(35.60)	
Primary school and below	683	(8.70)	294	(13.80)	156	(8.20)	127	(6.70)	106	(5.40)	
Mother’s education level (*n*, %)											<0.001^**^
College or higher	1,262	(16.10)	208	(9.80)	250	(13.20)	351	(18.70)	453	(23.20)	
High school	2,180	(27.70)	478	(22.40)	537	(28.40)	540	(28.70)	625	(32.00)	
Junior high school	3,244	(41.30)	967	(45.40)	830	(43.80)	750	(39.90)	697	(35.70)	
Primary school and below	1,176	(15.00)	479	(22.50)	277	(14.60)	241	(12.80)	179	(9.20)	
Family Computer and Internet situation (*n*, %)											<0.001^**^
Both	7,042	(89.60)	1,788	(83.90)	1,697	(89.60)	1,731	(92.00)	1,826	(93.40)	
One of the above	281	(3.60)	104	(4.90)	71	(3.70)	52	(2.80)	54	(2.80)	
Neither	539	(6.90)	240	(11.30)	126	(6.70)	99	(5.30)	74	(3.80)	
Accommodation (*n*, %)											<0.001^**^
No	5,553	(70.60)	1,342	(62.90)	1,330	(70.20)	1,389	(73.80)	1,492	(76.40)	
Yes	2,309	(29.40)	790	(37.10)	564	(29.80)	493	(26.20)	462	(23.60)	
Diet pattern score, mean (SD)											<0.001^**^
GDR-healthy score	37.46	(7.21)	36.19	(7.67)	36.39	(6.99)	37.66	(6.63)	39.69	(6.91)	
GDR-limit score	12.47	(4.07)	13.67	(4.87)	12.31	(3.74)	11.96	(3.57)	11.81	(3.57)	
Overall GDR score	24.99	(7.22)	22.52	(7.26)	24.08	(6.84)	25.70	(6.56)	27.88	(7.04)	

a Data are presented as mean (SD) for continuous measures, and *n* (%) for categorical measures, ^*^p < 0.05, ^**^p < 0.001.

**Figure 3:**
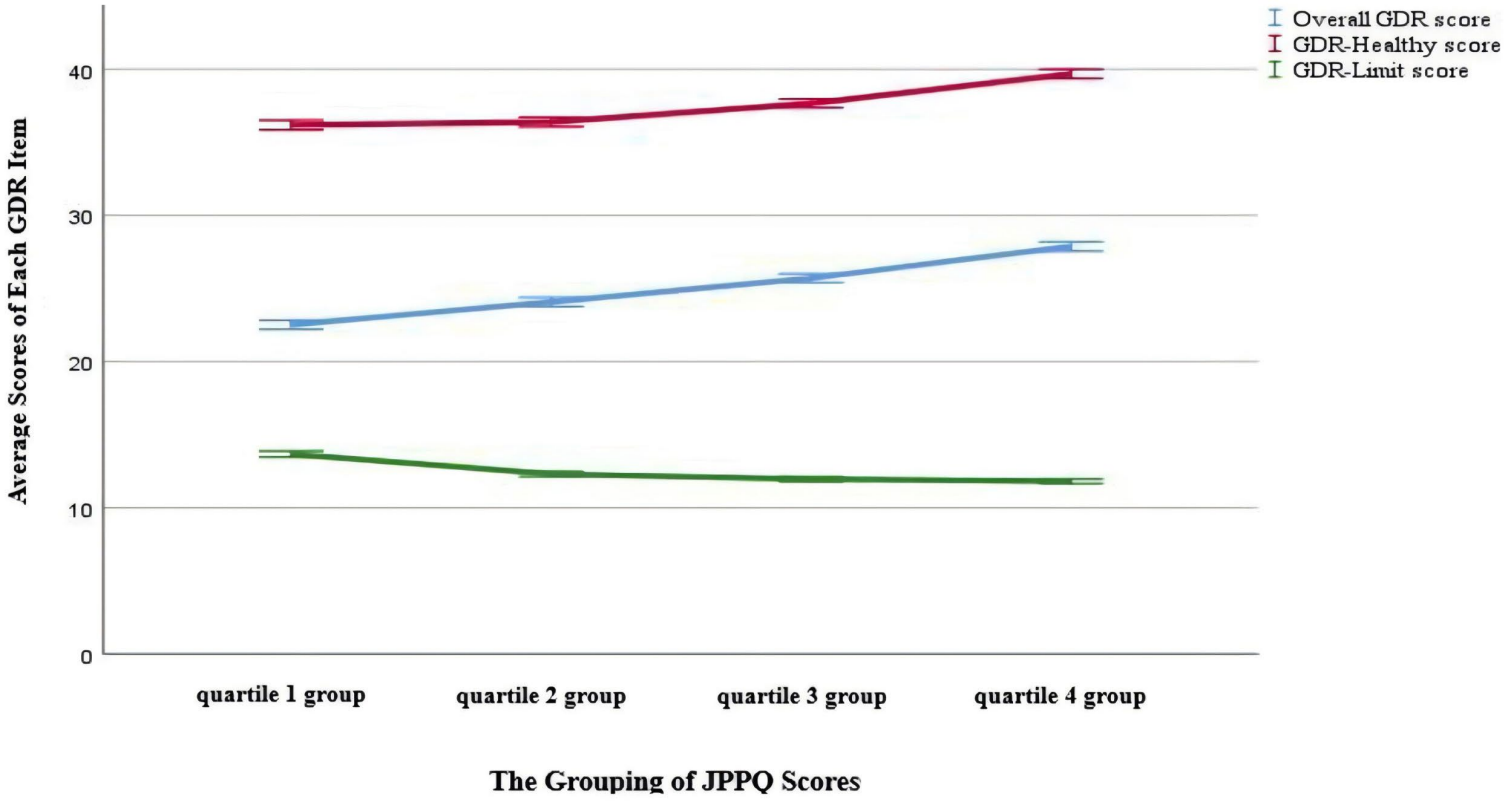
Relationship between participants’ performance on different GDR scores (GDR-Healthy, GDR-Limit, and Overall GDR) and their psychosocial profiles.

Additionally, 80.50% of adolescents were in the middle family wealth level. The vast majority of fathers (73.80%) and mothers (69.00%) had education levels of junior high or senior high school. Participants in the highest psychosocial profiles subgroup (the quartile 4 group) had a GDR-healthy score of 39.69 ± 6.91, a GDR-limit score of 11.81 ± 3.57 and an overall GDR score of 27.88 ± 7.04. Figure 3 shows the relationship between participants’ performance on different GDR scores (GDR-healthy, GDR-limit, and overall GDR) and their psychosocial status.

### Association between adolescents’ psychosocial profiles and healthy dietary scores

There was a positive association between psychosocial profile scores and healthy dietary pattern scores. In the fully adjusted model, across the quartiles of psychosocial profiles score, the regression coefficient (95%CI) for the healthy dietary score was 0.00, 1.33 (0.90–1.77), 2.84 (2.41–3.28), and 4.89 (4.44–5.34), respectively (*p* for trend < 0.001) (Table 2). The psychosocial score was positively associated with the GDR-heathy score but inversely associated with the GDR-limit score (Supplementary material S3). Good self-perception, harmonious peer relationships, and no or little experience with school bullying were positively associated with healthy dietary behaviors (Supplementary Figure S1).

**Table 2:** The relationship between quartiles of adolescents’ JPPQ score and healthy dietary pattern score.

	Model 1^a^				Model 2^b^				Model 3^c^			
	β	95% CI		*p*-value	β	95% CI		*p*-value	β	95% CI		*p*-value
Quartile 2 vs. Quartile 1	1.56	1.13	1.99	<0.001^**^	1.37	0.93	1.80	<0.001^**^	1.33	0.89	1.76	<0.001^**^
Quartile 3 vs. Quartile 1	3.18	2.75	3.61	<0.001^**^	2.89	2.46	3.33	<0.001^**^	2.84	2.40	3.27	<0.001^**^
Quartile 4 vs. Quartile 1	5.35	4.93	5.78	<0.001^**^	4.95	4.50	5.40	<0.001^**^	4.89	4.44	5.34	<0.001^**^

a Model 1 was the univariate model without adjustment for covariates.

b Model 2 was adjusted for demographic covariates.

c Model 3 was additionally adjusted for the family computer network situation.

All models refer to the first quartile range ^*^p < 0.05, ^**^p < 0.001.

### Stratified analysis according to demographic characteristics and family computer and Internet situation

In subgroup analyses, the association between the psychosocial profile scores and overall GDR was stronger in families without computers and the Internet than in those with these resources (*p*-value for interaction). No associations between psychological scores and other sociodemographic factors were found (Table 3).

**Table 3:** Subgroup analyses of the relationship between quartiles of the JPPQ score and the healthy dietary pattern score.

	Quartiles of JPPQ score^a^							
	Quartile 1	Quartile 2		Quartile 3		Quartile 4		*p* value for interaction
Sex								0.346
Boys	0.00	1.73	(1.08–2.38)	2.89	(2.24–3.54)	5.02	(4.37–5.66)	
Girls	0.00	1.00	(0.43–1.57)	2.81	(2.23–3.39)	4.76	(4.17–5.35)	
Nationality								0.327
Han	0.00	1.30	(0.86–1.73)	2.85	(2.41–3.29)	4.91	(4.48–5.35)	
Other	0.00	2.70	(−0.90–6.31)	2.63	(−1.03–6.29)	4.34	(0.44–8.23)	
Accommodation								0.677
No	0.00	1.14	(0.62–1.65)	2.63	(2.12–3.15)	4.76	(4.24–5.27)	
Yes	0.00	1.59	(0.80–2.39)	3.14	(2.32–3.97)	4.93	(4.09–5.77)	
Residence								0.528
Urban	0.00	1.01	(0.37–1.65)	2.44	(1.80–3.07)	4.43	(3.80–5.05)	
Rural	0.00	1.54	(0.95–2.12)	3.12	(2.52–3.73)	5.22	(4.59–5.85)	
Only child								0.187
Yes	0.00	1.93	(1.01–2.86)	3.34	(2.45–4.23)	4.83	(3.96–5.70)	
No	0.00	1.16	(0.68–1.65)	2.66	(2.16–3.16)	4.93	(4.42–5.44)	
Father’s education level								0.835
Primary school and below	0.00	0.54	(−0.88–1.95)	2.86	(1.35–4.37)	4.38	(2.79–5.98)	
Junior high school	0.00	1.19	(0.55–1.83)	2.90	(2.23–3.57)	4.93	(4.25–5.62)	
High school	0.00	1.29	(0.51–2.07)	2.52	(1.74–3.31)	4.99	(4.22–5.75)	
College or higher	0.00	2.09	(0.92–3.26)	3.24	(2.16–4.31)	4.78	(3.72–5.85)	
Mother’s education level								0.287
Primary school and below	0.00	1.35	(0.28–2.43)	2.76	(1.64–3.89)	4.19	(2.94–5.44)	
Junior high school	0.00	1.20	(0.56–1.85)	2.91	(2.25–3.58)	5.24	(4.56–5.92)	
High school	0.00	0.99	(0.15–1.83)	2.04	(1.20–2.88)	4.43	(3.62–5.25)	
College or higher	0.00	1.86	(0.61–3.11)	3.74	(2.56–4.91)	4.91	(3.78–6.05)	
Family economic status								0.983
Poor	0.00	1.34	(0.10–2.58)	3.12	(1.83–4.40)	4.70	(3.37–6.03)	
Medium	0.00	1.35	(0.88–1.82)	2.83	(2.35–3.30)	4.97	(4.49–5.45)	
Good	0.00	0.91	(−1.29–3.12)	2.42	(0.54–4.30)	3.96	(2.23–5.69)	
Family computer and Internet situation								0.011^*^
None	0.00	3.19	(1.48–4.90)	5.16	(3.29–7.03)	6.65	(4.61–8.69)	
One of the above	0.00	0.83	(−1.26–2.93)	4.59	(2.24–6.94)	3.78	(1.46–6.09)	
Both	0.00	1.15	(0.69–1.60)	2.56	(2.11–3.02)	4.73	(4.27–5.18)	

a Models adjusted for demographic characteristics and family computer and Internet situations.

^*^p < 0.05, ^**^p < 0.001.

## Discussion

In our study focusing on adolescents in Shandong, China, it was observed that good psychosocial profiles were linked to higher overall GDR scores, indicating a greater likelihood of adopting healthy dietary behaviors, especially among those with families without computers and the Internet. This healthy dietary pattern was characterized by a high intake of fruits, vegetables, fish, and dairy products [35].

### Comparison with evidence in the literature

Consistent with our findings, recent systematic reviews and meta-analyses have shown that poor psychological status is linked to unhealthy eating patterns (41). The use of psychological medications may affect dietary quality (42). Rodgers et al. (43) reported that adolescent boys and girls frequently exhibit eating disorder behaviors due to psychological issues, low mood, and other psychological factors associated with social media, as highlighted in a biopsychosocial model. Another study in Jordan found that adolescents who experienced negative peer pressure and self-esteem issues had a high prevalence of eating disorders. These disorders are frequently associated with unhealthy behaviors, such as smoking and alcohol consumption (44). This result aligns with our findings. During adolescence, harmonious peer relationships facilitate the development of healthy dietary habits; conversely, they can also lead to unhealthy behaviors. People with celiac disease who have good self-perception tend to eat healthily, and this positive self-perception is linked to a better quality of life among Chilean schoolchildren (45). Roy et al. (46) found that young adults who often buy and consume food on campus have worse diet quality scores compared to their counterparts, and approaches to improve the campus food environment may improve young adults’ diet quality.

Several mechanisms may explain the observed negative association between adherence to healthy dietary patterns and low psychosocial profile scores. Reward and hedonic mechanisms within the body play a significant role in food choices. The sight of tasty fruits and vegetables can reduce stress levels and trigger the release of hormones such as growth hormone-releasing peptides, insulin, and leptin, which promote healthy eating habits (47). Previous research found that adhering to a healthy dietary pattern can reduce the risk of psychosocial issues by increasing serum folate and vitamin B12 levels (48). Additionally, the potential reduction in psychosocial issues through the activation of the body’s reward mechanisms could serve as a strong incentive for adopting healthy dietary behaviors, mechanistically validating the relationship between psychological states and healthy dietary behaviors.

In subgroup analyses, the association between the psychosocial profile score and the overall GDR score was stronger among families without computers and the Internet than those with computers and the Internet. Consistent with our results, Marques et al. (49) defined a healthy lifestyle as including daily physical activity, limited screen time to less than 2 h, and a balanced consumption of vegetables and fruits. However, the widespread use of screens in contemporary society often promotes a sedentary lifestyle, causing individuals to spend prolonged periods in inactivity. Extended exposure to screens—whether televisions, computers, tablets, or smartphones—disrupts the natural rhythm of daily physical activity and fosters dependence on online activities, which can quickly escalate into addiction. This digital addiction, marked by an uncontrollable urge to check notifications, scroll through social media, or engage in gaming, significantly reduces both the time and motivation available for physical exercise. The results of the analysis of the 2010 Health Behavior in School-Aged Children International Survey Database (HBSC) revealed a linear decline in the prevalence of healthy behaviors from early adolescence to age 15 across 37 countries and territories. Specifically, the adolescents who used the Internet for hours in the study showed a notably reduced likelihood of adopting a healthy lifestyle compared with those with low screen contact. The Internet offers dual benefits: it facilitates social communication and fosters positive peer relationships among young individuals. However, it also introduces “cyberbullying,” a new form of school bullying that poses additional risks to the well-being of young people (50).

With the rapid development of Internet technology, the role of correct and healthy Internet use in the healthy development of young people cannot be ignored (50, 51). Nevertheless, in the case of universal use of social media, the personality traits of only children are diluted, peer relationship conflicts are weakened, and the psychological status develops in a favorable trend. In addition, a review suggested that AI-derived chat technology will facilitate the process of healthy behaviors among users (51). This result was contrary to the study’s findings that the existence of computer networks can encourage healthy eating habits. We deduce that in China, where education policies often involve confiscating media devices such as cell phones, adolescents may exhibit excessive usage once they return home. This behavior, commonly referred to as “revenge behavior,” arises as they feel the need to compensate for the restricted use during school hours. Consequently, this overuse of media devices at home can disrupt healthy eating behaviors and potentially reverse the positive dietary habits encouraged during school (52).

In summary, adolescents’ psychosocial profiles are closely linked to dietary behaviors. Numerous studies have demonstrated a bidirectional relationship between eating behaviors and psychological status (41, 53). The strengths of the study include a relatively large sample of adolescents from 100 schools in both urban and rural areas and the use of validated tools. Furthermore, we were able to adjust for potential confounders, such as personal characteristics (age, sex, residence, nationality, whether the individual is an only child, and whether they attend a boarding school) and family circumstances (family wealth, home computer network situation, and parental education level).

### Limitations

We also acknowledge the following limitations in our study: (a) dietary pattern scores are based on self-reported 1-week diets, which may be subject to recall bias and other bias; (b) the inability to measure family wealth accurately; and (c) the cross-sectional study design, which does not allow for establishing causality.

## Conclusion

A higher psychosocial score was associated with a higher likelihood of maintaining a healthy dietary pattern among adolescents in Shandong. However, no interaction was found between participants’ basic characteristics and their psychosocial profiles. The association between psychosocial characteristics and healthy dietary behaviors remained consistent across various demographic factors, including age, sex, residence, nationality, parental education level, family wealth, living conditions, and only-child status. The ownership of a computer and access to the Internet modified the association between the psychosocial profile and the healthy dietary score. Further research through in-depth mechanistic and cohort studies is required to explore the role of psychological factors in shaping eating patterns and behaviors.

## Data Availability

The data presented in the study are deposited in the the Population Health Data Archive (PHDA) repository, accession link https://doi.org/10.12213/11.A0031.202107.209.V1.0.
