# Supplementary material for: Psychosocial profiles influencing healthy dietary behaviors among adolescents in Shandong Province, China: a cross-sectional study
Source: Front Nutr. 2024 Sep 19;11:1418950. doi: 10.3389/fnut.2024.1418950 (PMC11448453; doi:10.3389/fnut.2024.1418950)
Supplement: Supplementary file 1 [file Data_Sheet_1.ZIP › supplementary materials/supplementary material 1.docx]

**Psychosocial Profiles Influencing Healthy Dietary Behavior Among Adolescents in Shandong Province of China：A Cross-Sectional Study**

| **Appendix 1 Dietary Status** | | | | | |
| --- | --- | --- | --- | --- | --- |
| **Preface**  **Number** | **Encoding** | **Data items contain**  **Yi** | **Data class**  **Type** | **Data item standard** | **Instructions;** |
|  | stulD | Individual number | Int type | 6 digits, as a unique identification  Code | This questionnaire contains 9 questions |
| 1 | q0401a | Number of diets last week | Int type | Value range: 1-5, respectively: more than once a day; About once a day; About once every other day; 1-2 times; 0 times | Green leafy vegetables |
| 2 | q0401b |  | Int type |  | Red and orange vegetables (carrots, tomatoes, etc.) |
| 3 | q0401c |  | Int type |  | Potatoes (excl. french fries, fried potatoes, or potato chips) |
| 4 | q0401d |  | Int type |  | Other vegetables |
| 5 | q0401e |  | Int type |  | Potato (sweet potato, yam, taro) |
| 6 | q0401f |  | Int type |  | Fruit |
| 7 | q0401g |  | Int type |  | Soy products (tofu, soy products) |
| 8 | q0401h |  | Int type |  | Chicken, pork, beef or other meats, fresh  Cooked or in a dish or soup) |
| 9 | q0401i |  | Int type |  | Fish, shrimp or other seafood |
| 10 | q0401j |  | Int type |  | Eggs |
| 11 | q0401k |  | Int type |  | Milk (milk, yogurt, etc.) |
| 12 | q0401l |  | Int type |  | Processed meats (ham sausage, meat sausage, red sausage, etc.) |
| 13 | q0401  m |  | Int type |  | Instant noodles |
| 14 | q0401n |  | Int type |  | Western fast food (refers to from McDonald's, KFC, Pizza Hut  Or other foods bought at Western fast food restaurants, including hamburgers, fried chicken, fish sticks, French fries, and pizza  Cake. |
| 15 | q0401o |  | Int type |  | Sweetened drinks, such as soda, sweetened milk or sweetened  Juice drinks |
| 16 | q0401p |  | Int type |  | Snacks and desserts with sugar or salt, such as cakes, cakes  Dried, candy, potato chips or shrimp chips; |
| 17 | q0401q |  | Int type |  | Fried food |
