# Supplementary material for: Psychosocial profiles influencing healthy dietary behaviors among adolescents in Shandong Province, China: a cross-sectional study
Source: Front Nutr. 2024 Sep 19;11:1418950. doi: 10.3389/fnut.2024.1418950 (PMC11448453; doi:10.3389/fnut.2024.1418950)
Supplement: Supplementary file 1 [file Data_Sheet_1.ZIP › supplementary materials/supplementary material 2.docx]

*Psychosocial Profiles Influencing Healthy Dietary Behavior Among Adolescents in Shandong Province of China：A Cross-Sectional Study*

| **Appendix 2 Psychological Cognition of Junior High School Students** | | | | | |
| --- | --- | --- | --- | --- | --- |
| **Preface**  **Number** | **Encoding** | **Data item**  **Meaning** | **Data item standard** | **Instructions;** |  |
|  | stulD | Personal editorial  Number | 6 digits as a unique identifier | This questionnaire contains 5 questions. See  'Post-numbered questionnaire' | Dimension |
| 1 | q050  1a | 0501 | Value range: 1-5, from low to high indicates the degree of appropriateness of the description to itself  1. Never; 2. Very few; 3. Sometimes; 4. Often; 5. Always/Often | A. On the whole, I have a lot to be proud of | Self  Cognition |
| 2 | q050  1b | 0602 |  | b. I do things as well as most people do | Self  Cognition |
| 3 | q050  1c | 0603 |  | c. I am good in many ways | Self  Cognition |
| 4 | q050  1d | 0604 |  | d. I'm as good as most people | Self  Cognition |
| 5 | q050  1e | 0605 |  | e. Other people think I'm a good person | Self  Cognition |
| 6 | q050  1f | 0606 |  | f. I try to do everything well | Self  Cognition |
| 7 | q050  2a | 0607 | Value range: 1-5, from low to high indicates the degree of appropriateness of the description to itself  1. Completely inconsistent; 2. Not very consistent; 3. Sometimes conform; 4. It is more consistent; 5. Fits perfectly | A. I make new friends easily at school | peer  Relation |
| 8 | q050  2b | 0608 |  | b. I can't find anyone to talk to | peer  Relation |
| 9 | q050  2c | 0609 |  | c. I like to study with other students | peer  Relation |
| 10 | q050  2d | 0610 |  | d. I have many friends | peer  Relation |
| 11 | q050  2e | 0611 |  | e. I can find friends when I need them | peer  Relation |
| 12 | q050  2f | 0612 |  | f. I get along well with other students | peer  Relation |
| 13 | q050  2g | 0613 |  | g. No one will help me when I need help | peer  Relation |
| 14 | q050  2h | 0614 |  | h. I don't get along with other classmates | peer  Relation |
| 15 | q050  3 | 0615 | Value range: 1-6, where 1 means never, 6 means more than once a week | Being belittled or bullied by classmates; | Campus  Bullying |
| 16 | q050  4 | 0616 |  | Be despised by the teacher | Campus  Bullying |
| 17 | q050  5 | 0617 |  | Be belittled by people outside school; | Campus  Bullying |
| 18 | City | City | Value range: 1-17; Representing Ji Nan, Qing Dao, Zi Bo, Zao Zhuang, Dong Ying, Yantai, Weifang, Jining, Tai' an, Weihai, Rizhao, Bin Zhou, De Zhou, Liao Cheng, Lin Yi, He Ze,  Lai wu. |  |  |
