# Supplementary material for: Psychosocial profiles influencing healthy dietary behaviors among adolescents in Shandong Province, China: a cross-sectional study
Source: Front Nutr. 2024 Sep 19;11:1418950. doi: 10.3389/fnut.2024.1418950 (PMC11448453; doi:10.3389/fnut.2024.1418950)
Supplement: Supplementary file 1 [file Data_Sheet_1.ZIP › supplementary materials/supplementary material 3.docx]

**Psychosocial Profiles Influencing Healthy Dietary Behavior Among Adolescents in Shandong Province of China：A Cross-Sectional Study**

Table 1 Association between psychosocial profiles score and GDR-healthy score

|  | Model 1^a^ | | | | Model 2^b^ | | | |
| --- | --- | --- | --- | --- | --- | --- | --- | --- |
| characteristic | OR | [95% CI] | | P | OR | [95% CI] | | P |
| psychosocial profiles score |  |  |  |  |  |  |  |  |
| Q2 vs. Q1 | 1.27 | 1.20 | 1.35 | <0.001 | 1.23 | 1.16 | 1.30 | <0.001 |
|  |  |  |  |  |  |  |  |  |
| Q3 vs. Q1 | 1.43 | 1.35 | 1.51 | <0.001 | 1.37 | 1.29 | 1.45 | <0.001 |
|  |  |  |  |  |  |  |  |  |
| Q4 vs. Q1 | 1.53 | 1.44 | 1.61 | <0.001 | 1.46 | 1.38 | 1.55 | <0.001 |

^a^ Model 1 was the univariate model in which no covariates were adjusted.

b Model 2 was adjusted for demographic covariates and family computer and Internet situations.

Table 2 Association between psychosocial profiles score and GDR-limit score

|  | Model 1^a^ | | | | Model 2^b^ | | | |
| --- | --- | --- | --- | --- | --- | --- | --- | --- |
| characteristic | OR | [95% CI] | | P | OR | [95% CI] | | P |
| psychosocial profiles score |  |  |  |  |  |  |  |  |
| Q2 vs. Q1 | 0.90 | 0.85 | 0.94 | <0.001 | 0.87 | 0.83 | 0.92 | <0.001 |
|  |  |  |  |  |  |  |  |  |
| Q3 vs. Q1 | 0.89 | 0.84 | 0.94 | <0.001 | 0.86 | 0.81 | 0.92 | <0.001 |
|  |  |  |  |  |  |  |  |  |
| Q4 vs. Q1 | 0.70 | 0.66 | 0.74 | <0.001 | 0.70 | 0.66 | 0.74 | <0.001 |

^a^ Model 1 was the univariate model in which no covariates were adjusted.

^b^ Model 2 was adjusted for demographic covariates and family computer and Internet situations.

Figure 1

| 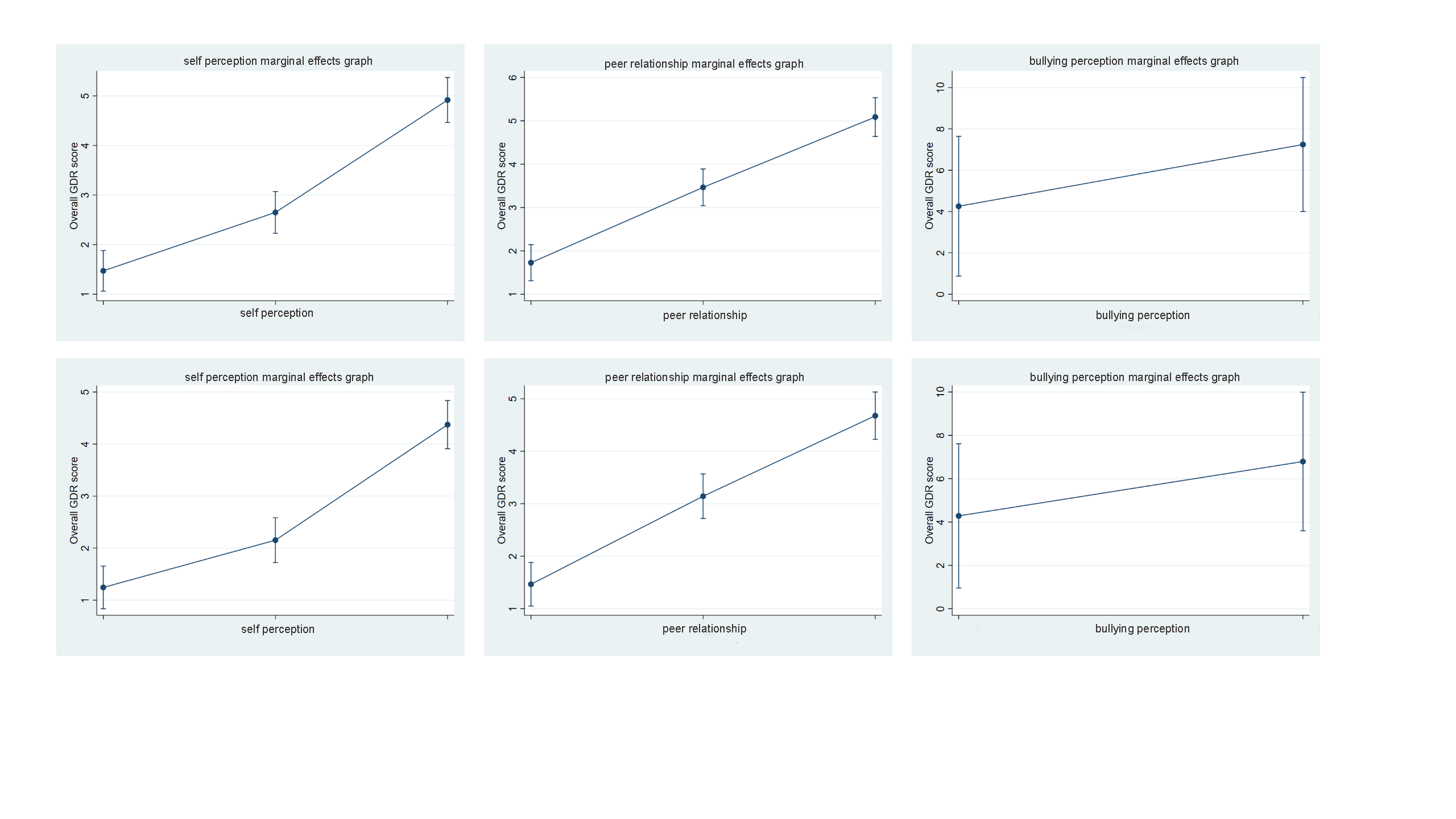  Three Dimensions of psychosocial profiles score related to dietary behavior.  Notes: the above image was he univariate model in which no covariates were adjusted and the below image was adjusted for demographic covariates and family computer and internet situations. |  |
| --- | --- |
|  |  |
|  |  |
|  |  |
